# Trends and hotspots of the neuroprotection of hypothermia treatment: A bibliometric and visualized analysis of research from 1992 to 2023

**DOI:** 10.1111/cns.14795

**Published:** 2024-06-12

**Authors:** Yang Zhang, Miaowen Jiang, Song Baoying, Yuan Gao, Yi Xu, Zhengfei Qi, Di Wu, Ming Li, Xunming Ji

**Affiliations:** ^1^ Department of Neurology Xuanwu Hospital, Capital Medical University Beijing China; ^2^ China‐America Institute of Neurology, Xuanwu Hospital, Capital Medical University Beijing China; ^3^ Beijing Institute for Brain Disorders, Capital Medical University Beijing China; ^4^ School of Instrumentation and Optoelectronic Engineering, Beihang University Beijing China; ^5^ Department of Neurosurgery Xuanwu Hospital, Capital Medical University Beijing China

**Keywords:** bibliometric analysis, brain injury, Citespace, hypothermia, ischemic stroke, neuroprotection

## Abstract

**Aim:**

Recent studies have extensively investigated hypothermia as a therapeutic approach for mitigating neural damage. Despite this, bibliometric analyses specifically focusing on this area remain scarce. Consequently, this study aims to comprehensively outline the historical framework of research and to pinpoint future research directions and trends.

**Methods:**

Articles spanning from 2003 to 2023, relevant to both “neuroprotection” and “hypothermia”, were sourced from the Web of Science Core Collection. The CiteSpace software facilitated a comprehensive evaluation and analysis of these publications. This analysis included examining the annual productivity, collaboration among nations, institutions, and authors, as well as the network of co‐cited references, authors and journals, and the co‐occurrence of keywords, and their respective clusters and trends, all of which were visualized.

**Results:**

This study included 2103 articles on the neuroprotection effects of hypothermia, noting a consistent increase in publications since 1992. The United States, the University of California System, and Ji Xunming emerged as the most productive nation, institution, and author, respectively. Analysis of the top 10 co‐cited publications revealed that seven articles focused on the effects of hypothermia in infants with hypoxic–ischemic encephalopathy, while three studies addressed cardiac arrest. Shankaran S and the journal *Stroke* were the most frequently co‐cited author and journal, respectively. Keyword cluster analysis identified ischemic stroke as the primary focus of hypothermia therapy historically, with cardiac arrest and neonatal hypoxic–ischemic encephalopathy emerging as current research foci.

**Conclusions:**

Recent studies on the neuroprotective effects of hypothermia in cardiac arrest and neonatal hypoxic–ischemic encephalopathy suggest that hypothermia may mitigate neural damage associated with these conditions. However, the application of hypothermia in the treatment of ischemic stroke remains confined to animal models and in vitro studies, with a notable absence of evidence from multicenter randomized controlled trials (RCTs). Further research is required to address this gap.

## Graphical abstract

This research utilizes bibliometric analysis to systematically evaluated the progress made in the topic of neuroprotection of hypothermia. Through this work, researchers can fully understand the research course and hot points related to the neuroprotective impact of hypothermia.

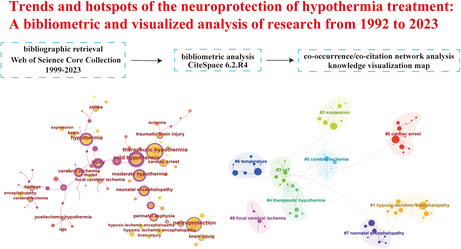

## 1 INTRODUCTION

The reduction of temperature as a preservation method for tissues is widespread in both the plant and animal kingdoms. In the realm of human medicine, organ cooling is recognized for its utility in transplantation, primarily because hypothermia reduces the metabolic demands of excised organs, thus extending their viability.^1^ Additionally, lowering temperatures decreases the accumulation of metabolic products following ischemia, which in turn minimizes the formation of harmful reactive oxygen species during reperfusion by limiting their reaction with oxygen. Furthermore, extensive research supports the efficacy of hypothermia in reducing cell death and inflammation. Therefore, hypothermia is a valuable strategy for mitigating ischemia–reperfusion (I/R) injury.^2^

The most sensitive organ in the human body to hypoxia is brain which is also more likely to be damaged by I/R injury. Hypothermia has been explored as a potential intervention to mitigate I/R injury due to its neuroprotective effects. At present, numerous studies have identified the protective effect of cooling brain on cerebral disease such as ischemic stroke,^3^, ^4^ cardiac arrest,^5^ neonatal hypoxic–ischemic encephalopathy,^6^ and so on. It is generally accepted that maintaining brain temperature between 32 and 36°C is optimal. However, the degree of neuroprotection hypothermia provides varies significantly across different diseases. Additionally, reducing body temperature below 34°C may cause severe systemic complications, including adverse effects on the cardiovascular, hematological, immune, and metabolic systems.^7^ Despite its potential, the underlying mechanisms of hypothermic neuroprotection remain incompletely understood. Given the promising prospects of hypothermia in neuroprotection and the existing gaps in knowledge, conducting a comprehensive literature review is crucial to discerning emerging trends and research focal points, thereby guiding further investigations in this area.

Bibliometric analysis, an expanding discipline within information science, provides comprehensive examination of publication characteristics over specified periods.^8^ This methodology enables researchers to assess a field both qualitatively and quantitatively, facilitating insights into prevailing research trends and hotspots.^9^, ^10^, ^11^ To date, according to our knowledge, no bibliometric studies have specifically addressed the topic of hypothermia's neuroprotective effects.

In this study, we utilized bibliometric analysis to evaluate the publication trends in the field of hypothermia's neuroprotective effects. The analysis encompassed the annual volume of publications, collaboration between country/region, affiliations, authors, citation metrics, and keyword frequencies. This systematic analysis aims to delineate the advancements and emerging focuses within this domain. The results provide researchers with a comprehensive overview of the trajectory and current hotspots in the study of hypothermia's neuroprotection.

## 2 METHODS

### 2.1 Search strategy

The Web of Science Core Collection (WoSCC) was selected as the primary database for data retrieval. A search strategy using topic searches (TS) for “neuroprotection OR neuroprotective” AND “hypothermia” was implemented to identify relevant studies. The inclusion criteria were limited to documents classified as “article” or “review.” Consequently, book chapters (*n* = 2), proceedings papers (*n* = 15), early access articles (*n* = 6), and retracted publications (*n* = 3) were excluded. The scope of the search was confined to literature published between January 1, 1992, and November 1, 2023. Non‐English publications were also omitted. After excluding non‐compliant and redundant entries, a total of 2103 original English articles met the inclusion criteria and were subjected to the final analysis.

### 2.2 Analysis tool

CiteSpace version 6.2.R4, developed by Professor Chaomei Chen, facilitated the analysis.^8^ The analysis settings were as follows: the time slice extended from January 1999 to November 2023, with each slice representing a 5‐year interval. Node types selected included country/region, institution, author, keyword, reference, cited author, and cited journal. *Selection* criteria alternated between g‐index (k = 25) and top *N* (*N* = 30), with cosine chosen as the link strength. Pruning options encompassed pathfinder, sliced networks, and merged networks.

**FIGURE 1:**
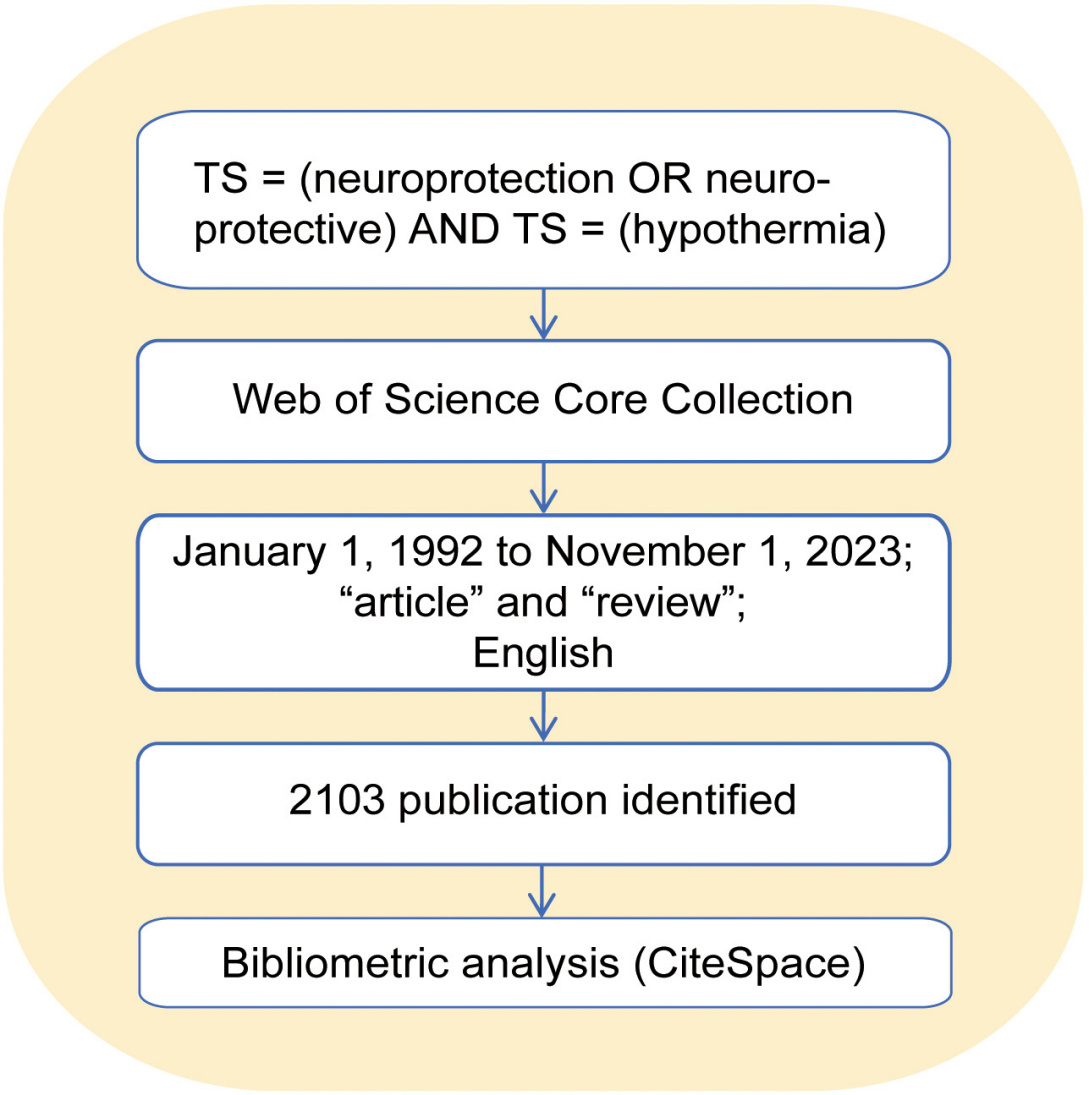
The flowchart illustrating the search strategy and analysis process for hypothermia neuroprotection.

The primary outputs included analysis of annual publication trends, cooperation between countries/regions, institutions, and authors, and co‐citation of references, authors, and journals. The analysis also covered co‐occurrence, clustering, and burst analysis of keywords. Node size was positively correlated with recurrence frequency. Nodes surrounded by an outer purple circle indicated high mediated centrality (≥0.1), denoting a pivotal and important connecting role. Links between nodes represented co‐occurrence and co‐cited relationships, with line thickness illustrating the strength of these relationships. For keyword cluster analysis, the log‐likelihood ratio test was applied. Figure 1 illustrates the search strategy and analysis process for hypothermia neuroprotection.

## 3 RESULTS

### 3.1 Annual quantity trend of publication

**FIGURE 2:**
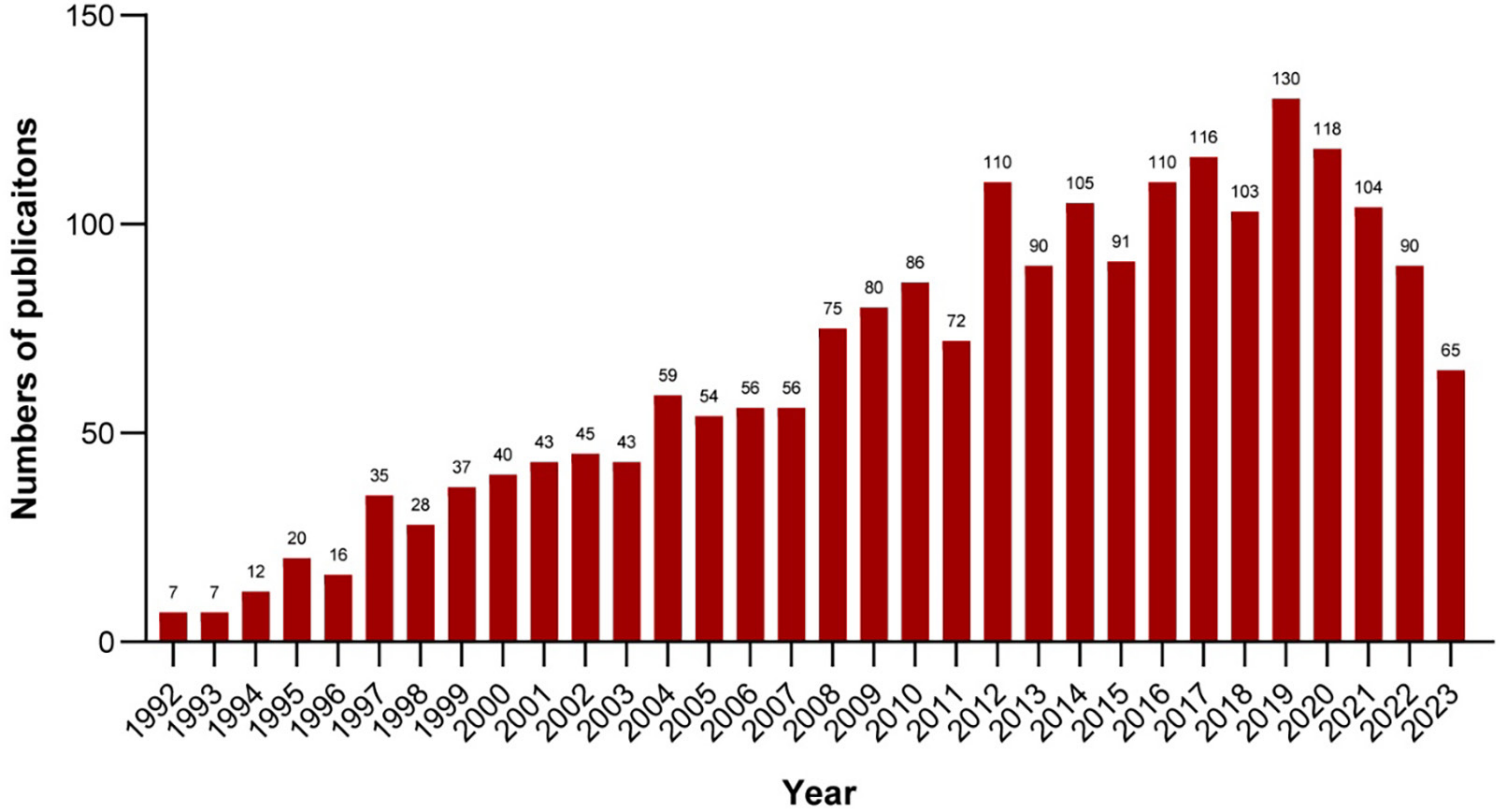
Annual quantity of publications on neuroprotection of hypothermia from 1992 to 2023.

The quantity of publications serves as an insightful indicator of the current state and potential future trajectory of a research field. As demonstrated in Figure 2, since the topic's emergence in 1992, the number of articles concerning the neuroprotection effects of hypothermia has increased steadily, reaching a peak in 2019 with 130 publications. Despite a subsequent decline in the volume of publications after 2019, the annual output has stabilized, maintaining approximately 100 publications annually from 2020 to 2022, with the exception of the incomplete data for 2023.

### 3.2 Analysis of collaborating countries/regions, institutions, and authors

**FIGURE 3:**
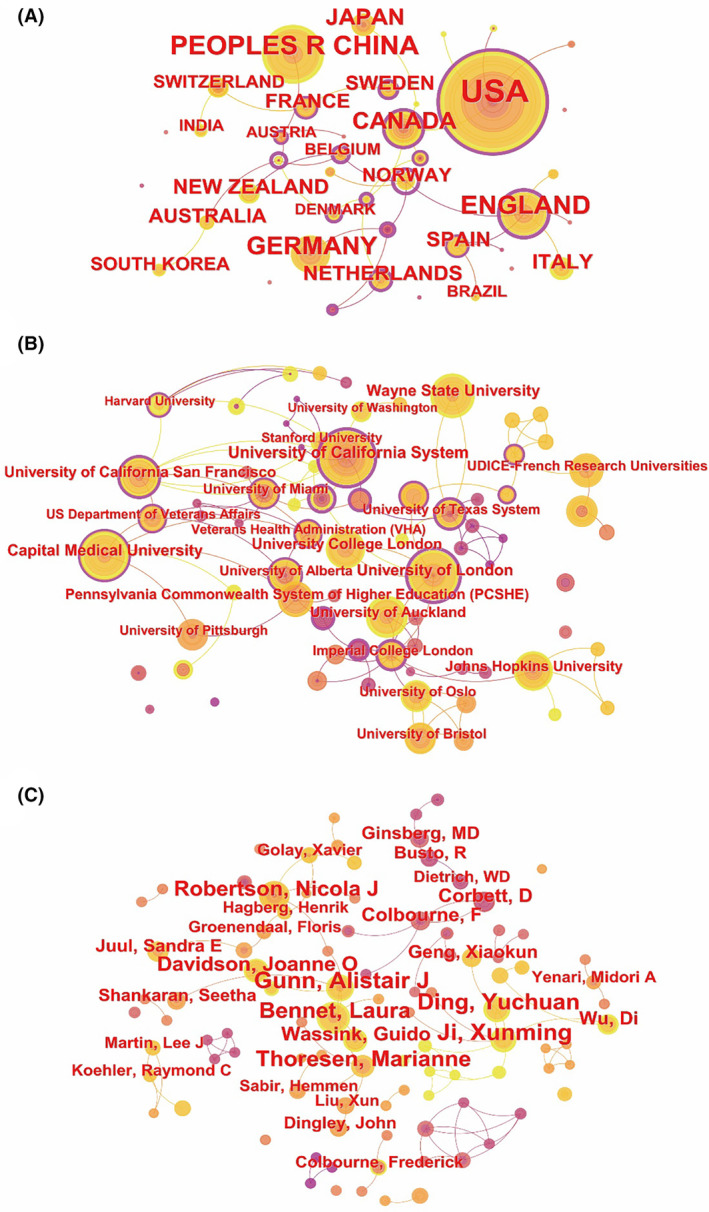
Collaboration networks for hypothermia neuroprotection. (A) Country/region collaboration. (B) Institution collaboration. (C) Author collaboration.

Co‐occurrence analysis was conducted to evaluate the collaborative relationships among countries/regions, institutions, and authors in the field of hypothermia neuroprotection. Initially, the analysis of countries/regions produced 42 nodes and 36 links, with the United States, China, and England being the most prolific, accounting for 39.4%, 13.7%, and 10.1% of all publications, respectively (Figure 3A and Table 1). Germany, Canada, Japan, Italy, New Zealand, Spain, and the Netherlands followed in publication count. Notably, England, Spain, and Canada demonstrated the highest centrality among the top 10 publishing countries, indicating robust collaborations with other nations (Figure 3A and Table 1). The institutional analysis revealed 106 nodes and 123 links. The University of California System led with 99 publications (4.7%) and the second‐highest centrality (0.46). The University of London followed with 83 publications (3.9%) and the highest centrality (0.49), underscoring these institutions' central roles in publishing and collaboration in this research area (Figure 3B and Table 1). Other prominent institutions included Wayne State University, Capital Medical University, University College London, University of California San Francisco, Johns Hopkins University, University of Auckland, Pennsylvania Commonwealth System of Higher Education, and University of Bristol. The author analysis comprised 154 nodes and 164 links. Ji Xunming, Gunn Alistair J, and Ding Yuchuan emerged as the top three most prolific authors (Figure 3C and Table 1). However, the centrality of the top 10 authors remained below 0.05, indicating relatively limited connectivity among them (Table 1).

**TABLE 1:** Top 10 productive countries/regions, institutions and authors.

Rank	Country/Region	Count (%)	Centrality	Institution	Count (%)	Centrality	Author	Count (%)	Centrality
1	USA	829 (39.4%)	0.15	University of California System	99 (4.7%)	0.46	Ji, Xunming	44 (2.1%)	0.02
2	China	288 (13.7%)	0.05	University of London	83 (3.9%)	0.49	Gunn, Alistair J	43 (2.0%)	0.04
3	England	213 (10.1%)	0.45	Wayne State University	80 (3.8%)	0.00	Ding, Yuchuan	40 (1.9%)	0
4	Germany	183 (8.7%)	0.05	Capital Medical University	76 (3.6%)	0.13	Thoresen, Marianne	39 (1.9%)	0.02
5	Canada	142 (6.8%)	0.24	University College London	65 (3.1%)	0.00	Bennet, Laura	36 (1.7%)	0.01
6	Japan	116 (5.5%)	0.00	University of California San Francisco	65 (3.1%)	0.37	Robertson, Nicola J	33 (1.6%)	0.03
7	Italy	76 (3.6%)	0.10	Johns Hopkins University	55 (2.6%)	0.08	Davidson, Joanne O	27 (1.3%)	0
8	New Zealand	67 (3.2%)	0.00	University of Auckland	54 (2.6%)	0.00	Wassink, Guido	23 (1.1%)	0
9	Spain	66 (3.1%)	0.33	Pennsylvania Commonwealth System of Higher Education	52 (2.5%)	0.03	Colbourne, F	21 (1.0%)	0
10	Netherlands	65 (3.1%)	0.23	University of Bristoly	47 (2.2%)	0.00	Corbett, D	21 (1.0%)	0

### 3.3 Analysis of co‐cited references, authors, and journals

**FIGURE 4:**
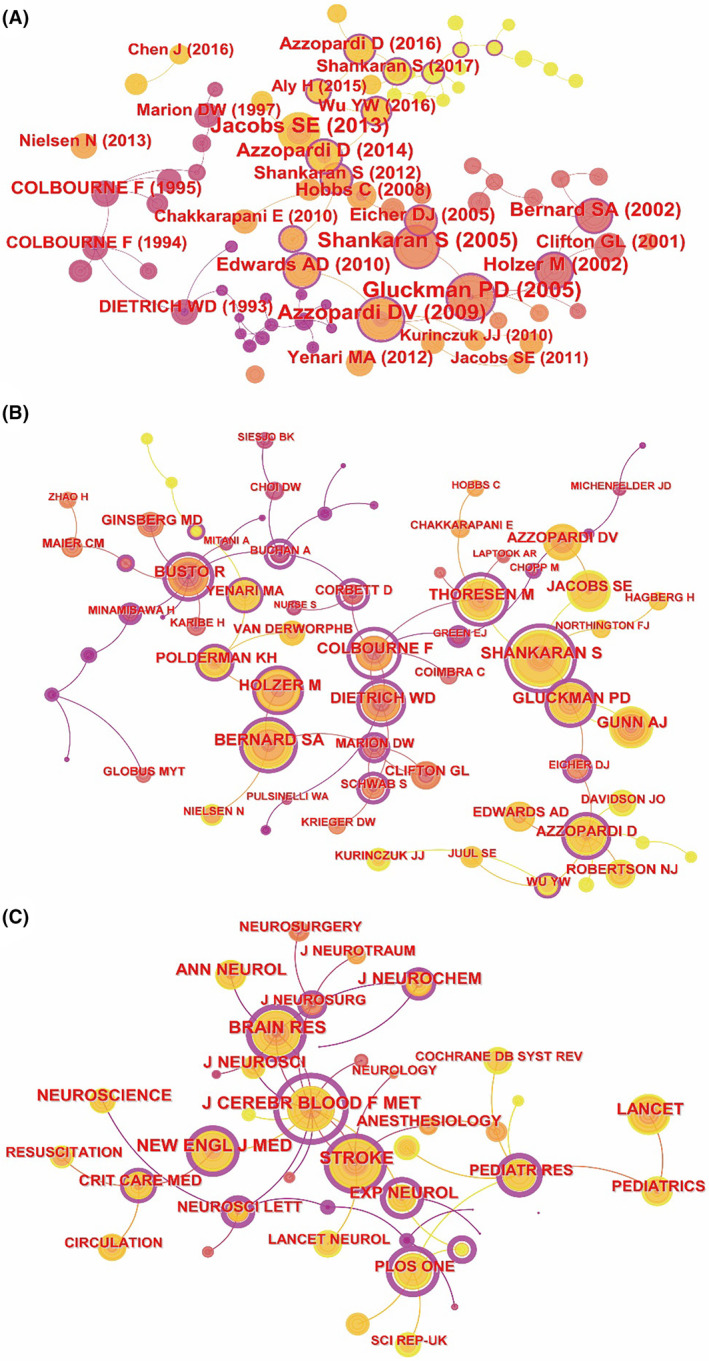
The co‐cited networks for hypothermia neuroprotection. (A) Co‐cited reference. (B) Co‐cited authors. (C) Co‐cited journals.

Co‐citation analysis involves the simultaneous citation of at least two references, authors, or journals by a single article, enabling the identification of influential literature, authors, and journals within specific fields. This analysis is depicted in Figure 4A, featuring 82 nodes and 79 links. Among the top 10 cited articles, seven addressed neonatal hypoxic–ischemic encephalopathy, while three focused on cardiac arrest, as detailed in Table 2.

**TABLE 2:** Top 10 co‐cited references.

Rank	Title	First Author	Journal	Year	Citation	Centrality	DOI
1	Selective head cooling with mild systemic hypothermia after neonatal encephalopathy: multicentre randomised trial^1^	Gluckman PD	*Lancet*	2005	103	0.03	10.1016/S0140‐6736(05)17946‐X
2	Whole‐body hypothermia for neonates with hypoxic‐ischemic encephalopathy^2^	Shankaran S	*N Engl J Med*.	2005	99	0.05	10.1056/NEJMcps050929
3	Moderate hypothermia to treat perinatal asphyxial encephalopathy^3^	Azzopardi DV	*N Engl J Med*.	2009	85	0.76	10.1056/NEJMoa0900854
4	Cooling for newborns with hypoxic ischaemic encephalopathy^4^	Jacobs SE	*Cochrane Database Syst Rev*.	2013	84	0.06	10.1002/14651858.CD003311.pub3
5	Mild therapeutic hypothermia to improve the neurologic outcome after cardiac arrest^5^	Holzer M	*N Engl J Med*.	2002	70	0.02	10.1056/NEJMoa012689
6	Treatment of comatose survivors of out‐of‐hospital cardiac arrest with induced hypothermia^6^	Bernard SA	*N Engl J Med*.	2002	68	0.00	10.1056/NEJMoa003289
7	Moderate hypothermia in neonatal encephalopathy: efficacy outcomes^7^	Eicher DJ	*Pediatr Neurol*.	2005	52	0.87	10.1016/j.pediatrneurol.2004.06.014
8	Effects of hypothermia for perinatal asphyxia on childhood outcomes^8^	Azzopardi D	*N Engl J Med*.	2014	52	0.67	10.1056/NEJMoa1315788
9	Neurological outcomes at 18 months of age after moderate hypothermia for perinatal hypoxic ischaemic encephalopathy: synthesis and meta‐analysis of trial data^9^	Edwards AD	*BMJ*.	2010	51	0.73	10.1136/bmj.c363
10	Targeted temperature management at 33 versus 36°C after cardiac arrest^10^	Nielsen N	*N Engl J Med*.	2013	46	0.02	10.1056/NEJMoa1310519

In the context of neonatal hypoxic–ischemic encephalopathy, the article “Selective head cooling with mild systemic hypothermia after neonatal encephalopathy: multicentre randomised trial,” authored by Gluckman et al and published in 2005, emerged as the most cited reference, receiving 103 citations and displaying a centrality of 0.03. Conversely, the publication with the highest centrality of 0.87 was “Moderate hypothermia in neonatal encephalopathy: efficacy outcomes,” authored by Eicher DJ et al. and also published in 2005.

Another focus was the neuroprotective effects of hypothermia on cardiac arrest. The most frequently cited paper on this subject is “Mild therapeutic hypothermia to improve the neurologic outcome after cardiac arrest” by Holzer M, published in 2002, with 70 citations (Table 2). However, the centrality values of three publications related to cardiac arrest were relatively low, at 0.02 and 0.00.

In the co‐cited author graph, which includes 70 nodes and 72 links (Figure 4B), Shankaran S emerged as the most cited author, receiving 449 citations with a centrality of 0.8. This author was followed by Bernard SA with 359 citations, Thoresen M with 324, Busto R with 311, Holzer M with 306, Gluckman PD with 285, Gunn AJ with 277, Colbourne F with 270, Dietrich WD with 270, and Azzopardi D with 243 citations. Notably, Colbourne F was identified as the most central author, with a centrality of 1.45 (Table 3).

**TABLE 3:** Top 10 cited authors and journals.

Rank	Cited authors	Citation	Centrality	Cited journals	Citation	Centrality
1	Shankaran S	449	0.8	Stroke	1399	0.36
2	Bernard SA	359	0.42	J Cerebr Blood F Met.	1357	1.62
3	Thoresen M	324	0.95	Brain Res.	1142	0.43
4	Busto R	311	0.68	New Engl J Med.	1115	0.26
5	Holzer M	306	0.32	J Neurosci.	820	0.00
6	Gluckman PD	285	0.59	Ann Neurol.	767	0.00
7	Gunn AJ	277	0.00	Lancet	714	0.00
8	Colbourne F	270	1.45	J Neurochem.	701	0.18
9	Dietrich WD	270	0.78	Neuroscience	680	0.00
10	Azzopardi D	243	0.49	Exp Neurol.	667	0.79

The network map of co‐cited journals, depicted in Figure 4C, comprises 43 nodes and 43 links. “*Stroke*” led in citation frequency with 1399 citations, while Journal of “*J Cerebr Blood F Med*” achieved the highest centrality score at 1.62, as shown in Table 3. Among the top 10 cited journals, “*New Engl J Med*” and “*Lancet*” emerged as leading publications within the medical community, with impact factors of 158.5 and 168.9, respectively.

### 3.4 Keywords analysis

**FIGURE 5:**
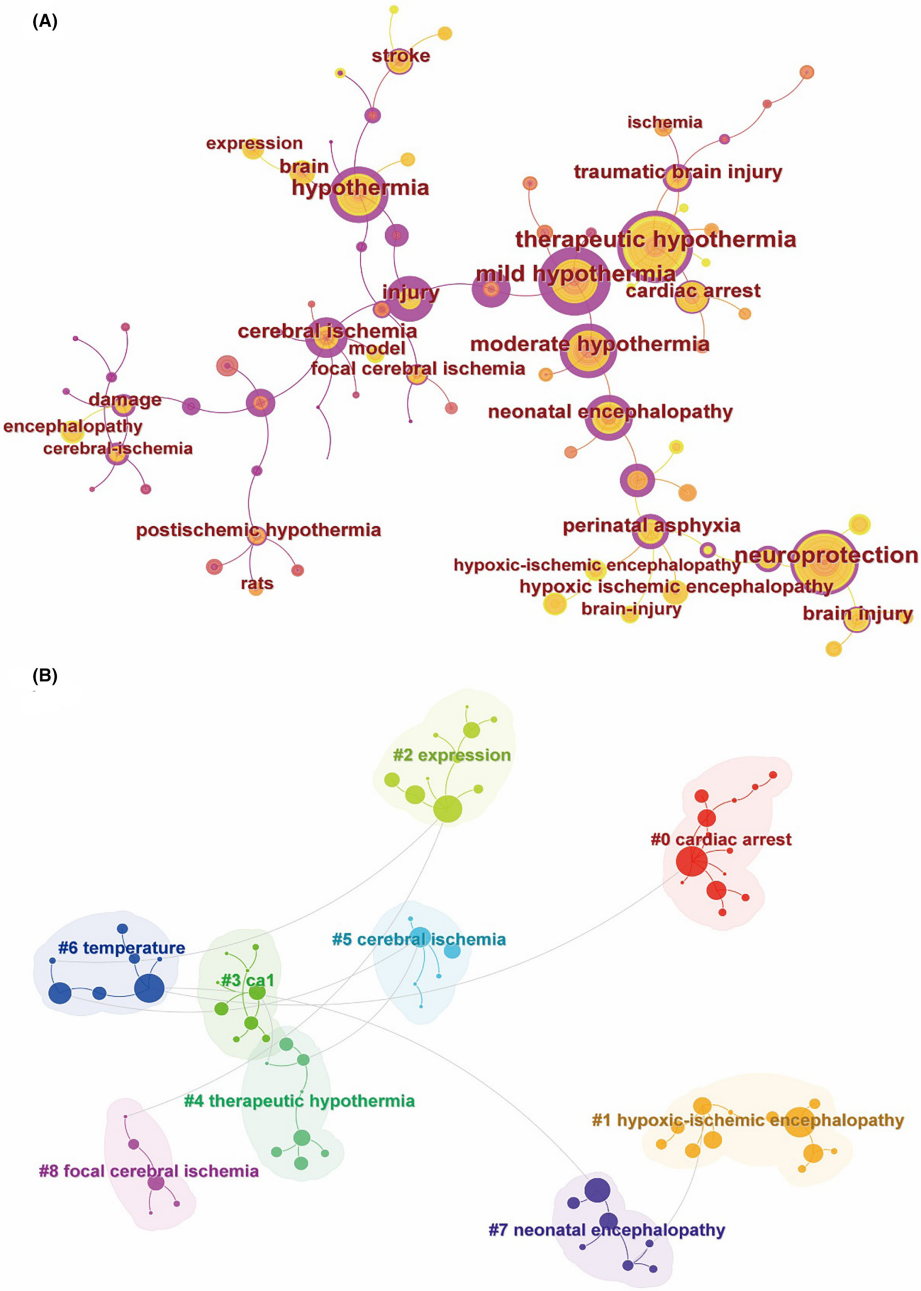
Keyword co‐occurring network map (A) and cluster network map (B).

Figure 5A depicts 78 nodes and 78 links, representing the co‐occurrence of keywords. Following keyword clustering, ischemic stroke (#5 and #8), cardiac arrest (#0), and neonatal hypoxic–ischemic encephalopathy (#1 and #7) emerged as the primary diseases targeted by hypothermia treatment (Figure 5B). Other clusters identified include #2 expression, #3 CA1, #4 therapeutic hypothermia, and #6 temperature.

**FIGURE 6:**
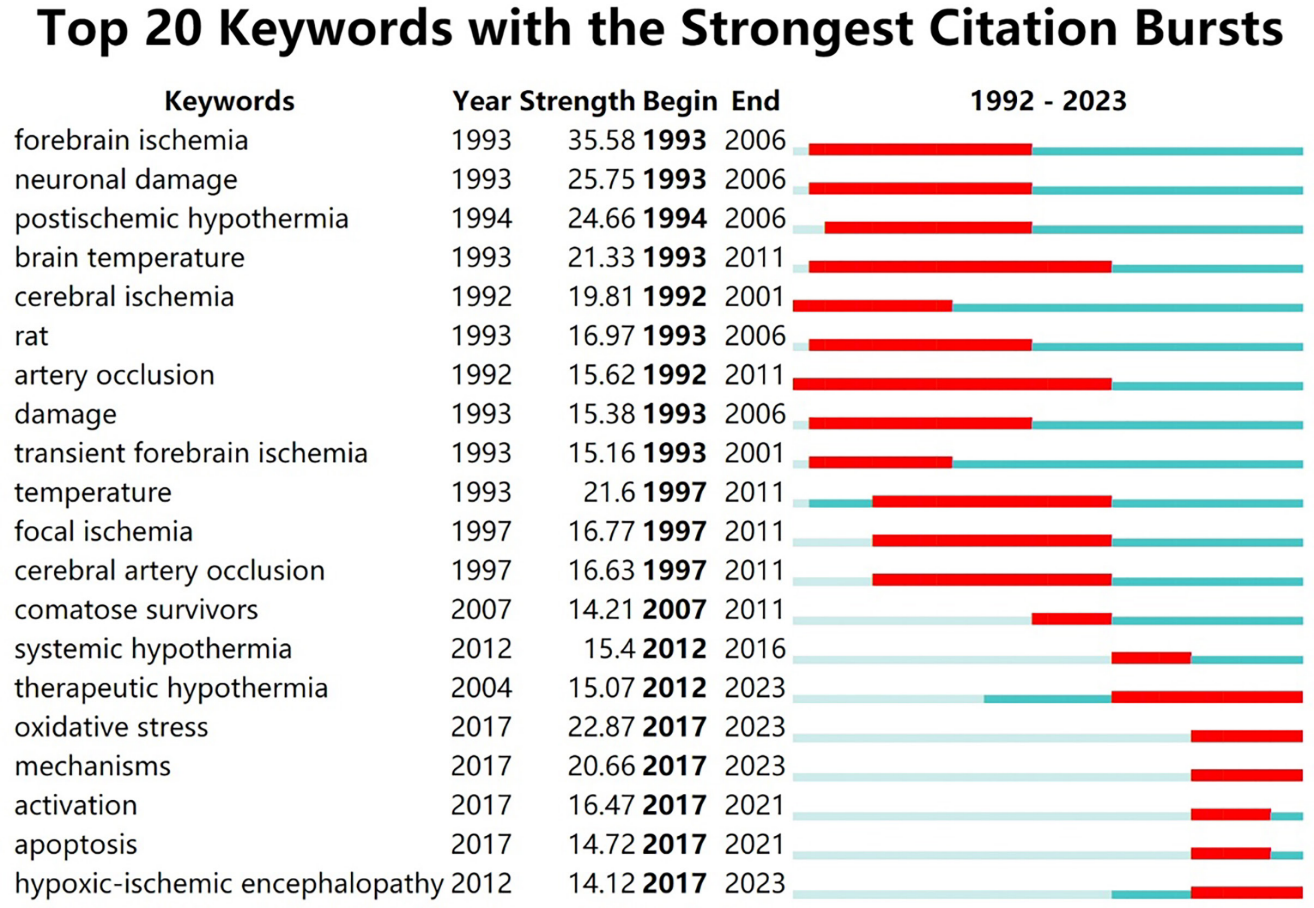
Top 20 keywords with the strongest citation bursts. The blue bar showed that the keyword was rarely cited, and the red bar indicated frequent citation

Figure 6 illustrates the top 20 keywords demonstrating the most significant citation bursts, with the foremost five being “forebrain ischemia,” “neuronal damage,” “postischemic hypothermia,” “oxidative stress,” and “brain temperature.” Notably, the terms “oxidative stress,” “mechanisms,” and “hypoxic–ischemic encephalopathy” showed pronounced bursts between 2021 and 2023, reflecting increased attention and advancement in these areas.

## 4 DISCUSSION

Bibliometric analysis systematically examines the research landscape, trends, and focal points within specific fields. In this study, CiteSpace software was utilized to assess the status of research on hypothermia neuroprotection. Over the past three decades, investigations into hypothermia neuroprotection have predominantly centered on randomized controlled trials (RCTs) for various neurological conditions.^1^, ^5^, ^11^ Additionally, research has explored neuroscience‐based mechanisms derived from animal or cellular studies,^12^ side effects associated with hypothermia,^13^ and the development of hypothermia methodologies.^14^

The publication volume on the neuroprotective effects of hypothermia has shown a steady increase over the first two decades, stabilizing during the period from 2012 to 2022 with an average of 106 publications annually. This trend underscores sustained interest in this research area for a considerable duration. Despite a noticeable decline in the annual publication count since 2019, the numbers remain relatively high. This pattern may reflect the established therapeutic benefits of hypothermia for specific conditions, which might reduce the necessity for further basic research. Nevertheless, numerous unresolved questions persist within the domain of hypothermic brain protection that warrant continued investigation.

In terms of publication volume, the United States of America leads significantly, contributing to 39.4% of the total publications in neuroprotection of hypothermia studies. Additionally, half of the top 10 productive institutions are based in the USA. Regarding centrality, while England tops the list of countries with the most published literature, Norway holds a more central position among all countries (0.95). This suggests that despite Norway's lower publication volume, researchers there are more inclined to collaborate internationally. Among the top 10 productive institutions, the University of California System and the University of London rank first and second, respectively, in both publication volume and centrality. Hence, these two institutions play pivotal roles in advancing this field.

Ji Xunming from Capital Medical University in China has the highest number of publications. He has long been dedicated to studying the protective effects of hypothermia on acute ischemic stroke and is currently focusing on targeted hypothermia in brain tissue via arterial routes. The top 10 most prolific authors exhibit low centrality, suggesting that the collaboration among them is not particularly close. This implies the necessity for increased communication among these authors in the future.

Among the top 10 cited references, 8 are multicenter RCT studies and 2 are meta‐analyses of RCTs, illustrating the widespread application of hypothermia therapy for nerve injury in clinical practice. For instance, the most referenced study on neonatal encephalopathy is a multicenter RCT assessing the impact of mild hypothermia post‐neonatal encephalopathy.^1^ Despite not meeting initial efficacy expectations for the mixed‐population infants, subgroup analysis revealed that brain cooling can safely enhance survival among infants with less severe amplitude‐integrated electroencephalography changes and without severe neurodevelopmental disorders. This study is also seminal in the realm of RCT studies on hypothermia treatment for neonatal hypoxic–ischemic encephalopathy. Another prominent multicenter RCT investigation is the most cited reference concerning cardiac arrest.^5^ This study suggests that therapeutic moderate hypothermia could reduce mortality and enhance neurologic outcomes in individuals successfully resuscitated following ventricular fibrillation‐induced cardiac arrest. All top 10 cited references concur that hypothermia confers benefits in cases of neonatal encephalopathy and cardiac arrest, reflecting a broad consensus on the efficacy of hypothermia in addressing these conditions.

According to the analysis of co‐cited authors, Shankaran^15^ emerges as the most frequently cited author, with a longstanding dedication to clinical research on systemic hypothermia for neonatal hypoxic–ischemic encephalopathy. Following closely is Bernard,^16^ whose focus lies in exploring hypothermic protection in cases of cardiac arrest. Moreover, the most centrally cited author is Colbourne^17^, ^18^ whose research predominantly delves into the hypothermic effects on focal ischemic stroke and intracerebral hemorrhage, These top 10 cited authors play pivotal roles in propelling the advancement of hypothermia neuroprotection. Therefore, future researchers aiming to delve deeper into this field should thoroughly examine their work. The Journal of Cerebr Blood F Met, serving as a comprehensive neuroscience journal amalgamating both basic and clinical research, holds the position of the most central cited journal and ranks second in citation frequency. For instance, Lyden et al's^19^ study utilized an in vitro model to propose a scheme for optimizing therapeutic hypothermia following cerebral ischemia/reperfusion injury. Additionally, Wu et al^20^ documented the safety, feasibility, and efficacy of intra‐arterial selective cooling infusion for stroke patients undergoing mechanical thrombectomy. Hence, this journal demonstrates a keen interest in this domain and stands as an ideal platform for future scholars to disseminate their research.^19^

According to co‐occurrence keyword analysis, traumatic brain injury emerges as another significant focus in contemporary research on hypothermia treatment, alongside the diseases previously mentioned.^21^ Additionally, keyword clustering indicates that the ca1 region might serve as the primary protective area under hypothermic conditions.^22^ Examination of keyword bursts reveals that mechanisms constitute the current focal points of basic research into hypothermia‐induced neuroprotection,^23^ particularly in mitigating oxidative stress damage.^24^

An intriguing revelation from the historical analysis of keyword bursts is the enduring prominence of ischemic stroke as a research hotspot in this field. Despite its validation in animal models, the efficacy of hypothermia in ischemic stroke patients remains unproven by multicenter RCT. This underscores the need for heightened scrutiny and investigation into this phenomenon.

### 4.1 The future direction of hypothermic therapy for ischemic stroke

The failure of clinical translation in hypothermic neuroprotection for ischemic stroke stems from various factors, among which the side effects of hypothermia play a significant role. Whole‐body hypothermia presents a range of adverse effects, including shivering, infection, coagulation abnormalities, electrolyte imbalances, and hyperglycemia, all of which negatively impact patient prognosis. Therefore, achieving a balance between the benefits and adverse effects of hypothermic treatment becomes a crucial prognostic indicator for patients.^25^ While the entire brain experiences severe ischemic injury during cardiac arrest and neonatal encephalopathy, focal brain damage occurs following acute ischemic stroke. Consequently, patients with cardiac arrest and neonatal encephalopathy may derive greater benefits from whole‐brain hypothermia to making the benefits outweigh the harm of side effects than ischemic stroke patients. To enhance the efficacy of hypothermic treatment for ischemic stroke patients, minimizing side effects appears to be imperative.

Considering the risks associated with systemic hypothermia, the adoption of brain hypothermia emerges as a promising future direction. Utilizing ice caps represents the initial approach to selectively lower brain temperature.^26^ However, ice caps predominantly cool the cortex with limited impact on deeper brain tissues. An alternative technique involves transnasal cooling. The human nasal cavity boasts ample blood flow, a substantial mucosal surface area, and close proximity to cerebral circulation. Theoretically, brain cooling through the nasal cavity is expedited. Primary methods entail circulating cool water through the nasal passage and continuously administering a blend of coolant and oxygen.^27^ Given the proximity of the nasal cavity to the skull base, a combination of transnasal cooling and ice cap therapy appears more effective for brain cooling. These non‐invasive and selective modalities offer promising avenues for reducing brain temperature. In recent years, with the maturation and widespread adoption of intravascular thrombectomy technology, mounting evidence has underscored its favorable impact on patient outcomes.^28^ Consequently, for patients undergoing thrombectomy, intra‐arterial hypothermia emerges as a more expedient and targeted approach for reducing brain temperature.^29^

Continuous perfusion of a low‐temperature solution through a microcatheter placed in the intracranial artery can quickly reduce brain tissue temperature. Recent clinical studies all use saline as the cooling solution for a short cooling period because long‐term continuous infusion of saline can increase the burden on heart function and reduce the hematocrit.^30^, ^31^ In principle, while maintaining core temperature, the longer the selective low‐temperature time for brain tissue, the better neuroprotection could be obtained. Thus, drawn inspiration from extracorporeal membrane oxygenation technology, intra‐artery cooling autologous blood perfusion technology proposed by Ji xunming group seems to be the best way to resolve all existing concerns. This technology can draw blood from the femoral artery of the patient, cool the blood through extracorporeal circulation, and infuse it back into the brain through an intracranial arterial catheter, continuously selectively reducing focal brain temperature without increasing blood volume. At present, this technology has been validated for safety and neuroprotective effectiveness in primates.^32^ However, notably, this is only an animal study and must be clarified in further clinical validation.

In summary, given the potential adverse effects of systemic hypothermia, selective brain cooling appears to be a more appropriate approach for focal stroke patients. For patients at any stage following the onset of the disease, the use of ice caps combined with nasal hypothermia may represent the optimal therapeutic strategy for clinicians. Furthermore, for patients requiring thrombectomy, the future emphasis may lie in intra‐arterial cooling autologous blood perfusion technology post‐thrombectomy.

## 5 LIMITATION

This study still has some limitations. Firstly, there may be language and publication biases as we solely analyzed English papers from the Web of Science database. Secondly, relying solely on Citespace may introduce bias into the results due to the limitations of this single method. Lastly, due to constraints of space and research objectives, a comprehensive discussion on the neuroprotection hotspots of hypothermia is lacking.

## 6 CONCLUSION

This study presents the initial bibliometric and visual analysis of hypothermia's neuroprotective effects. Over the last 30 years, research output in this field has remained consistently high, with a notable shift in focus from ischemic stroke initially, to cardiac arrest, and finally to neonatal hypoxic–ischemic encephalopathy. Additionally, current research highlights mechanisms such as oxidative stress as significant areas of interest. Previous studies have shown hypothermia's potential in mitigating neural damage post‐cardiac arrest and in neonatal hypoxic–ischemic encephalopathy. However, the application of hypothermia in ischemic stroke treatment has primarily been explored in animal and in vitro studies, and there remains a dearth of evidence from multicenter RCT. Further investigation is warranted to bridge this research gap, with intra‐arterial cooling and autologous blood perfusion following thrombectomy emerging as potential future research avenues.

## AUTHOR CONTRIBUTIONS

**Yang Zhang:** Conceptualization; formal analysis; investigation; methodology; software; writing—original draft; **Miaowen Jiang:** Software; writing‐review and editing; **Baoying Song:** Software; **Yuan Gao:** Software; **Yi Xu:** Software; **Zhengfei Qi:** Investigation; **Di Wu:** Writing—review and editing; **Li Ming:** Methodology; writing—review and editing; **Xunming Ji:** Conceptualization; project administration; writing—review and editing.

## FUNDING INFORMATION

This project was supported by grants from the National Natural Science Foundation of China (82027802 and 82102220). 2019 Beijing Ten Million Talents Project (2019A36); Research Funding on Translational Medicine from Beijing Municipal Science and Technology Commission (Z221100007422023); Beijing Municipal Administration of Hospitals Clinical Medicine Development of Special Funding Support from Yangfan Project (YGLX202325); the Non‐profit Central Research Institute Fund of Chinese Academy of Medical (2023‐JKCS‐09); and Beijing Association for Science and Technology Youth Talent Support Program (BYESS2022081); Beijing Municipal Natural Science Foundation (7244510); Science and Technology Innovation Service Capacity Building Project of Beijing Municipal Education Commission (11000023T000002157177); Outstanding Young Talents Program of Capital Medical University (B2305).

## CONFLICT OF INTEREST STATEMENT

The authors declare that they have no competing interests.

## Data Availability

The data that support the findings of this study are available from the corresponding author upon reasonable request.

## References

[1] Gluckman PD , Wyatt JS , Azzopardi D , et al. Selective head cooling with mild systemic hypothermia after neonatal encephalopathy: multicentre randomised trial. Lancet. 2005;365(9460):663‐670. doi:10.1016/S0140-6736(05)17946-X 15721471

[2] Shankaran S , Laptook AR , Ehrenkranz RA , et al. Whole‐body hypothermia for neonates with hypoxic‐ischemic encephalopathy. N Engl J Med. 2005;353(15):1574‐1584. doi:10.1056/NEJMcps050929 16221780

[3] Azzopardi DV , Strohm B , Edwards AD , et al. Moderate hypothermia to treat perinatal Asphyxial encephalopathy. N Engl J Med. 2009;361(14):1349‐1358. doi:10.1056/NEJMoa0900854 19797281

[4] Jacobs SE , Berg M , Hunt R , Tarnow‐Mordi WO , Inder TE , Davis PG . Cooling for newborns with hypoxic ischaemic encephalopathy. Cochrane Database Syst Rev. 2013;2013(1):CD003311. doi:10.1002/14651858.CD003311.pub3 23440789 PMC7003568

[5] Holzer M , Cerchiari E , Martens P , et al. Mild therapeutic hypothermia to improve the neurologic outcome after cardiac arrest. N Engl J Med. 2002;346(22):549‐556. doi:10.1056/NEJMoa012689 11856793

[6] Bernard SA , Gray TW , Buist MD , et al. Treatment of comatose survivors of out‐of‐hospital cardiac arrest with induced hypothermia. N Engl J Med. 2002;346(8):557‐563. doi:10.1056/NEJMoa003289 11856794

[7] Eicher DJ , Wagner CL , Katikaneni LP , et al. Moderate hypothermia in neonatal encephalopathy: efficacy outcomes. Pediatr Neurol. 2005;32(1):11‐17. doi:10.1016/j.pediatrneurol.2004.06.014 15607598

[8] Azzopardi D , Strohm B , Marlow N , et al. Effects of hypothermia for perinatal asphyxia on childhood outcomes. N Engl J Med. 2014;371(2):140‐149. doi:10.1056/NEJMoa1315788 25006720

[9] Edwards AD , Brocklehurst P , Gunn AJ , et al. Neurological outcomes at 18 months of age after moderate hypothermia for perinatal hypoxic ischaemic encephalopathy: synthesis and meta‐analysis of trial data. BMJ. 2010;340:c363. doi:10.1136/bmj.c363 20144981 PMC2819259

[10] Nielsen N , Wettersley J , Cronberg T , et al. Targeted temperature management at 33°C versus 36°C after cardiac arrest. N Engl J Med. 2013;369(23):2197‐2206. doi:10.1056/NEJMoa1310519 24237006

[11] Cheng Z , Ding Y , Rajah GB , et al. Vertebrobasilar artery cooling infusion in acute ischemic stroke for posterior circulation following thrombectomy: rationale, design and protocol for a prospective randomized controlled trial. Front Neurosci. 2023;17:1149767. doi:10.3389/fnins.2023.1149767 37113154 PMC10126519

[12] Yenari MA , Han HS . Neuroprotective mechanisms of hypothermia in brain ischaemia. Nat Rev Neurosci. 2012;13(4):267‐278. doi:10.1038/nrn3174 22353781

[13] Wu D , Chen J , Zhang X , Ilagan R , Ding Y , Ji X . Selective therapeutic cooling: to maximize benefits and minimize side effects related to hypothermia. J Cereb Blood Flow Metab. 2022;42(1):213‐215. doi:10.1177/0271678X211055959 34670442 PMC8721772

[14] Li M , Gao Y , Jiang M , et al. Dual‐sized hollow particle incorporated fibroin thermal insulating coatings on catheter for cerebral therapeutic hypothermia. Bioact Mater. 2023;26:116‐127. doi:10.1016/j.bioactmat.2023.02.022 36879558 PMC9984786

[15] Thayyil S , Montaldo P , Krishnan V , et al. Whole‐body hypothermia, cerebral magnetic resonance biomarkers, and outcomes in neonates with moderate or severe hypoxic‐ischemic encephalopathy born at tertiary care centers vs other facilities: a nested study within a randomized clinical trial. JAMA Netw Open. 2023;6(5):e2312152. doi:10.1001/jamanetworkopen.2023.12152 37155168 PMC10167567

[16] Bernard SA , Smith K , Finn J , et al. Induction of therapeutic hypothermia during out‐of‐hospital cardiac arrest using a rapid infusion of cold saline the RINSE trial (rapid infusion of cold Normal saline). Circulation. 2016;134(11):797‐805. doi:10.1161/circulationaha.116.021989 27562972

[17] Baker TS , Durbin J , Troiani Z , et al. Therapeutic hypothermia for intracerebral hemorrhage: systematic review and meta‐analysis of the experimental and clinical literature. Int J Stroke. 2022;17(5):506‐516. doi:10.1177/17474930211044870 34427479

[18] Liddle LJ , Kalisvaart ACJ , Abrahart AH , Almekhlafi M , Demchuk A , Colbourne F . Targeting focal ischemic and hemorrhagic stroke neuroprotection: current prospects for local hypothermia. J Neurochem. 2022;160(1):128‐144. doi:10.1111/jnc.15508 34496050

[19] Lyden PD , Lamb J , Kothari S , Toossi S , Boitano P , Rajput PS . Differential effects of hypothermia on neurovascular unit determine protective or toxic results: toward optimized therapeutic hypothermia. J Cereb Blood Flow Metab. 2019;39(9):1693‐1709. doi:10.1177/0271678X18814614 30461327 PMC6727141

[20] Wu C , Zhao W , An H , et al. Safety, feasibility, and potential efficacy of intraarterial selective cooling infusion for stroke patients treated with mechanical thrombectomy. J Cereb Blood Flow Metab. 2018;38(12):2251‐2260. doi:10.1177/0271678X18790139 30019993 PMC6282221

[21] Trieu C , Rajagopalan S , Kofke WA , Cruz Navarro J . Overview of hypothermia, its role in neuroprotection, and the application of prophylactic hypothermia in traumatic brain injury. Anesth Analg. 2023;137(5):953‐962. doi:10.1213/ANE.0000000000006503 37115720

[22] Zhao J , Xia C , Tang Y , Wan H . Role of PERK‐mediated pathway in the effect of mild hypothermia after cerebral ischaemia/reperfusion. Eur J Clin Investig. 2023;53(10):e14040. doi:10.1111/eci.14040 37337313

[23] Lin JQ , Khuperkar D , Pavlou S , et al. HNRNPH1 regulates the neuroprotective cold‐shock protein RBM3 expression through poison exon exclusion. EMBO J. 2023;42(14):e113168. doi:10.15252/embj.2022113168 37248947 PMC10350819

[24] Yang L , Dong Y , Wu C , et al. Effects of prenatal photobiomodulation treatment on neonatal hypoxic ischemia in rat offspring. Theranostics. 2021;11(3):1269‐1294. doi:10.7150/thno.49672 33391534 PMC7738878

[25] Wu L , Wu D , Yang T , et al. Hypothermic neuroprotection against acute ischemic stroke: the 2019 update. J Cereb Blood Flow Metab. 2020;40(3):461‐481. doi:10.1177/0271678X19894869 31856639 PMC7026854

[26] Diprose WK , Morgan CA , Wang MT , et al. Active conductive head cooling of normal and infarcted brain: a magnetic resonance spectroscopy imaging study. J Cereb Blood Flow Metab. 2022;42(11):2058‐2065. doi:10.1177/0271678X221107988 35707879 PMC9580175

[27] Chen X , Xu S , Li M , Wu D , Ji X . Transnasal cooling: new prospect of selective hypothermia in acute ischemic stroke. J Cereb Blood Flow Metab. 2023;44:310‐312. doi:10.1177/0271678X231211726 37898106 PMC10993875

[28] Nogueira RG , Jadhav AP , Haussen DC , et al. Thrombectomy 6 to 24 hours after stroke with a mismatch between deficit and infarct. N Engl J Med. 2018;378(1):11‐21. doi:10.1056/NEJMoa1706442 29129157

[29] Chen J , Liu L , Zhang H , et al. Endovascular hypothermia in acute ischemic stroke: pilot study of selective intra‐arterial cold saline infusion. Stroke. 2016;47(7):1933‐1935. doi:10.1161/STROKEAHA.116.012727 27197848 PMC4927369

[30] Wan Y , Tian H , Wang H , Wang D , Jiang H , Fang Q . Selective intraarterial hypothermia combined with mechanical thrombectomy for acute cerebral infarction based on microcatheter technology: a single‐center, randomized, single‐blind controlled study. Front Neurol. 2023;14:1039816. doi:10.3389/fneur.2023.1039816 36873429 PMC9978520

[31] Tian H , Wan Y , Zhang H , Zuo J . Interrupted intraarterial selective cooling infusion combined with mechanical thrombectomy in patients with acute ischemic stroke: a prospective, nonrandomized observational cohort study. J Neurosurg. 2023;139(4):1083‐1091. doi:10.3171/2023.2.JNS222542 36964733

[32] Chen J , Xu S , Lee H , et al. Hypothermic neuroprotection by targeted cold autologous blood transfusion in a non‐human primate stroke model. Sci Bull. 2023;68(14):1556‐1566. doi:10.1016/j.scib.2023.06.017 37391345

